# Stimulants for the Control of Hedonic Appetite

**DOI:** 10.3389/fphar.2016.00105

**Published:** 2016-04-25

**Authors:** Alison S. Poulton, Emily J. Hibbert, Bernard L. Champion, Ralph K. H. Nanan

**Affiliations:** ^1^Sydney Medical School Nepean, The University of SydneyPenrith, NSW, Australia; ^2^Charles Perkins Centre Nepean, The University of SydneyPenrith, NSW, Australia

**Keywords:** appetite suppressants, dexamphetamine, obesity, reward deficiency, phentermine, hedonic appetite

## Abstract

The focus of this paper is treatment of obesity in relation to the management of hedonic appetite. Obesity is a complex condition which may be potentiated by excessive reward seeking in combination with executive functioning deficits that impair cognitive control of behavior. Stimulant medications address both reward deficiency and enhance motivation, as well as suppressing appetite. They have long been recognized to be effective for treating obesity. However, stimulants can be abused for their euphoric effect. They induce euphoria via the same neural pathway that underlies their therapeutic effect in obesity. For this reason they have generally not been endorsed for use in obesity. Among the stimulants, only phentermine (either alone or in combination with topiramate) and bupropion (which has stimulant-like properties and is used in combination with naltrexone), are approved by the United States Food and Drug Administration (FDA) for obesity, although dexamphetamine and methylpenidate are approved and widely used for treating attention deficit hyperactivity disorder (ADHD) in adults and children. Experience gained over many years in the treatment of ADHD demonstrates that with careful dose titration, stimulants can be used safely. In obesity, improvement in mood and executive functioning could assist with the lifestyle changes necessary for weight control, acting synergistically with appetite suppression. The obesity crisis has reached the stage that strong consideration should be given to adequate utilization of this effective and inexpensive class of drug.

## Introduction

The obesity epidemic has been attributed to the easy availability of high energy, palatable foods, together with an increasingly sedentary lifestyle. The solution to obesity should be a simple matter of balancing energy intake to requirement, but unfortunately this approach has had limited success and the prevalence of obesity continues to rise. Treatment of obesity has a cost, but could result in massive savings via long-term disease prevention.

The costs of obesity include the costs of treating the medical complications, the days of work missed and disability payments. The three main treatment modalities are lifestyle interventions, bariatric surgery or medication. A variety of psychological treatments have been trialed for assisting with making the required lifestyle changes (McElroy et al., 2015a). Success with lifestyle intervention depends on the individual's motivation and many cannot sustain the effort (Grodstein et al., 1996), limiting the cost-effectiveness of this modality. Bariatric surgery has the drawbacks of expense and surgical complications (Dixon, 2014), with ongoing costs of supplements to prevent nutritional deficiencies (Lopes et al., 2015). However, it has been calculated that the cost of surgery can be offset by savings in medication costs within 2–7 years, with those with existing obesity complications having the potential for the greatest savings (Lopes et al., 2015). Using more complex modeling, bariatric surgery appears highly cost-effective, with an average gain of 2.9 years of health at a relatively low cost of $9000 per year (Michaud et al., 2012). The benefits are less clear for medication as outcomes can be more variable. However, pharmacological treatment to help make necessary lifestyle changes more achievable would appear an attractive option, particularly if used as an early intervention. This would potentially involve more treatment-years and result in more modest benefits to a greater number of people. The cost-effectiveness of such treatment would be highly dependent on the cost of the drug. The cost of developing a new drug has been estimated at $2.6 billion (Avorn, 2015), which has to be recovered before the patent expires. This gives a substantial economic advantage to generic prescribing of older drugs.

One main target of pharmacotherapy is appetite suppression. Appetite control may be considered to have two components; homeostatic and hedonic. The homeostatic component has the essential function to maintain daily energy requirement for sustaining bodily functions such as body temperature, circulation, breathing, and the energy required for movement. Gut hormones like cholecystokinin, ghrelin, glucagon-like peptide-1, and peptide YY regulate the inclination to feed, having an immediate effect on sensations of hunger or satiety (Cummings and Overduin, 2007). Leptin reflects fat mass and acts over a longer time-frame to suppress the appetite as fat stores increase (Saad et al., 1998). Insulin, glucagon-like peptide-1, and cortisol have a regulatory role in energy balance and utilization (Osto et al., 2007). The hormonal homeostatic mechanisms adjust in response to weight loss with changes that increase the appetite and promote weight regain (Sumithran et al., 2011). However, eating is not simply a matter of meeting daily energy requirements but is also associated with pleasure. This leads to an additional hedonic drive to eat.

## Hedonic appetite

Hedonic appetite may be considered as a continuum, extending from the normal situation of individuals enjoying some foods more than others, through comfort eating which may occur when the mood is low, to the final extreme situation in which an individual appears to be addicted to food as to a drug of addiction.

## Neural mechanisms

### Reward mechanisms

The hedonic component of appetite is mediated by central reward mechanisms and has the ability to override homeostatic satiety mechanisms (Volkow et al., 2008). The enjoyment associated with eating is attributed to activation of the dopamine mediated reward pathway in the limbic region of the brain (Wang et al., 2009; DiLeone et al., 2012). The endocannabinoid and opioid systems are also involved in central appetite control, relating to food palatability, and interacting with mesolimbic reward pathway and reinforcing the motivation for food seeking (Volkow et al., 2008). The mesolimbic pathway communicates with the nucleus accumbens, which is involved in reward and appetite control. Wang et al. have shown reduced dopamine D_2_ receptor density in individuals with obesity (Wang et al., 2001). Although the resulting decreased dopaminergic activity could explain vulnerability to obesity, such that more food is required to obtain satisfaction (D'Addario et al., 2014), overeating of palatable food may itself lead to a reduction in the D_2_ receptors and promote compulsive eating (DiLeone et al., 2012). This suggests that a blunting of the reward mechanisms and associated desire for highly palatable food may be both a cause and a consequence of obesity. A similar situation pertains with drugs of abuse which may initially be taken for pleasure, subsequently progressing to craving, and diminished satisfaction due to desensitization of the reward mechanisms (Volkow et al., 2008). In each case the repetition leads to similar changes in the reward circuitry and associated reductions in the experience of pleasure for other activities, together with conditioning to stimulus related cues and compulsive consumption (Kenny et al., 2013).

The above mechanisms suggest that the enjoyment of palatable food may be pursued to excess, ultimately developing into a self-perpetuating cycle of craving and compulsive eating that is independent of hunger and which may manifest as binge eating (Ziauddeen et al., 2012; Gautron et al., 2015). This end-point may have contributing factors such as individual susceptibility (perhaps associated with low D_2_ receptors) and environmental factors (such as food availability) and lack of enjoyment or reward from other facets of life. An observational study showing that individuals who suffer from obesity may be prone to addiction (Reslan et al., 2014) supports the notion of a common susceptibility. The idea of obesity as an addiction is not new. The German word for obesity is “fettsucht” or “fat addiction.” Of course, putative food addiction differs from addiction to drugs as food is essential to life and there is no chemical withdrawal syndrome (Ziauddeen et al., 2012).

Although eating excessively for pleasure may progress to dissatisfaction, craving, and possible food addiction, this does not always happen. It has been suggested that some individuals may have under activity of their intrinsic reward mechanisms, resulting in reward seeking behavior that can be excessive and maladaptive. This has been termed reward deficiency syndrome (Downs et al., 2013). Dissatisfaction associated with a state of reward deficiency could be associated with low mood, which the individual might address with comfort eating. This could underpin the known association of obesity with depression (Dawes et al., 2016). This process could occur at a subtle level, with imperceptible discrepancies in the balance between energy intake and utilization over time leading to obesity.

This sensation of reward associated with food and other pleasurable stimuli is important for motivating animals to fulfill the basic functions that are necessary for the survival of the individual and the species. At a more subtle level, experiencing adequate satisfaction in the daily tasks of life helps to maintain a positive mood and outlook (Catalino and Fredrickson, 2011). Therefore, eating may be a strategy for mood regulation, with food restriction resulting in irritability.

### Executive functions

Excessive hedonic appetite may be overcome by the executive functions of the prefrontal cortex exerting behavioral control for curbing disinhibited eating. This requires consistent motivation. Individuals lacking sufficient executive control may find it harder to combat obesity, due to their greater difficulty in making decisions that involve delaying gratification (Davis et al., 2010). This has been supported by a study that showed lower levels of activation of the prefrontal cortex in response to food in obese non-dieters when compared to successful dieters (DelParigi et al., 2007). Individuals with obesity could therefore be excessively reward seeking and they could also have deficits in the executive functions associated with motivation and cognitive control of behavior. However, as with the reward mechanisms, obesity can itself lead to impaired executive functioning and behavioral control. One obvious manifestation of this is in the repeated relapses that may follow well-reasoned decisions to keep to a diet (Volkow et al., 2016).

Similar reward and executive functioning deficits also typify ADHD (Poulton and Nanan, 2014), perhaps the main difference being that in obesity the reward seeking and executive functioning deficits, such as impulsive behavior and poor motivation, result in excessive food intake. In both conditions these two types of predisposing deficit would be summative in their effects.

## Implications for therapy—stimulants

If an important biological mechanism is deficient to the extent that health is compromised, it is logical to try to treat the deficiency medically, targeting the pathways involved. Stimulants activate reward mechanisms by interacting with amine transporter systems, decreasing the synaptic reuptake of dopamine and noradrenaline (Madras et al., 2005), and to a lesser extent serotonin (Kuczenski and Segal, 1997) so that neurotransmission is enhanced. This results in mundane tasks appearing more rewarding (Volkow et al., 2004). Stimulants are also associated with appetite suppression, which correlates with the increased activity of the mesolimbic reward pathway and elevated extracellular dopamine the nucleus accumbens (Rowley et al., 2000). Therefore, stimulants address the reward deficit, which may rectify the compulsion to eat excessively and could also explain their positive effect on mood (Martin et al., 1971). However, stimulants have the additional effect of a dose-related improvement in executive functioning, hence their value in ADHD. It has been shown with methylphenidate that this improvement rises to a maximum and then declines once the optimal dose has been exceeded (Gamo et al., 2010). When treated with stimulants, children with ADHD are able to concentrate more consistently and are more efficient at task completion (MTA Cooperative Group, 1999). They are also more able to think rationally and consider the consequences of their actions.

The ability of the stimulants to target both the reward deficiency and the executive functioning deficits suggests that they are a logical treatment for obesity. At higher doses stimulants are associated with adverse effects on mood, progressing to nervousness, depression and emotional irritability, with reversible paranoia, and psychosis developing with further dose increases (Griffith et al., 1972). Stimulants are also associated with elevations in heart rate and blood pressure due to their sympathomimetic effects, which limits their use in individuals with cardiovascular disease (Stiefel and Besag, 2010). The practice of dose titration to maximize behavioral improvement and minimize adverse effects may explain their safety record in the treatment of ADHD (Merkel, 2010).

Although under activity of the reward pathway can lead to dissatisfaction and low mood, too much stimulation can be addictive and stimulants are recognized as drugs of abuse. The ability of stimulants to increase extracellular dopamine correlates not only with their therapeutic effect in ADHD and obesity but also with their ability to induce euphoria, which can be addictive (Volkow and Swanson, 2003). The reinforcing sensation of euphoria correlates with a rapid rate of dopamine receptor occupancy. As euphoria is dependent on the rate of absorption rather than the actual dose of stimulant, a dose of methylphenidate that might be therapeutic for oral administration, when given intranasally can produce euphoria comparable to that obtained using cocaine (Volkow and Swanson, 2003). Among the stimulants, methamphetamine is the most addictive as it rapidly accumulates in the brain, inducing euphoria even when taken orally (Fowler et al., 2008).

The first stimulant to be endorsed by the FDA for the treatment of obesity was methamphetamine in 1947 (United States Food and Drug Administration, 2012). In the 1950s and 1960s dexamphetamine was widely prescribed for a range of problems including obesity, depression, and poor motivation (Kiloh and Brandon, 1962). Although it was recognized that it might occasionally be taken as a habit to restore confidence it was generally considered safe even for long-term use (Editorial, BMJ, 1955). However, it came to light that some people were abusing dexamphetamine and had been fraudulently obtaining multiple prescriptions and having them dispensed by different pharmacies (Kiloh and Brandon, 1962). A few were admitted to hospital with psychosis and malnutrition, experiencing depression on drug withdrawal. Then the opinion suddenly turned against the stimulants for the treatment of obesity (United States Food and Drug Administration, 2012). Despite this, the stimulant phentermine has continued to be licensed for short term use in obesity and in combination with the anticonvulsant topiramate for long term use. Furthermore, bupropion, a drug used for treating depression and nicotine addiction, which has many similarities to the stimulants in its clinical effects, was licensed for treatment of obesity in combination with naltrexone in 2014 (United States Food and Drug Administration, 2014). Therefore, despite a widespread view that the use of stimulants for obesity is inappropriate, medications that appear remarkably similar in their effects and in their abuse potential are still being used. The use of the opiates for pain relief demonstrates that drugs that are highly addictive can be used therapeutically provided there are adequate safeguards. Similarly, the stimulants have continued to be prescribed for individuals with ADHD, a condition with recognized susceptibility to risky behavior including substance abuse (Molina et al., 2013).

The two main arguments against the stimulants is their abuse potential and their apparent loss of efficacy with time, once a new steady state has been reached after the initial weight loss. For example, the effect that stimulants have for enhancing reward could lead to inappropriate use, or potentiate addictive behavior or compulsions such as binge eating. However, lisdexamfetamine, a pro-drug for dexamphetamine, has been shown to be effective for reducing binge eating (McElroy et al., 2015b). Oral administration also reduces the risk as it limits the potential for euphoria and increases the probability of adverse gastrointestinal side effects such as nausea and fullness, which might be exacerbated by eating (DiLeone et al., 2012). Furthermore, stimulants are not associated with a physical withdrawal syndrome that promotes continuous use. Although stimulant abuse is a particular concern for people with risk factors for addiction, one very important distinction between individuals with obesity and those who have abused drugs is that food addiction does not involve any illegal activity. Therefore, such people could have strong reservations for using illegal or dishonest means for acquiring drugs.

When considering the advantages and disadvantages of the stimulants as outlined above, we consider the most appropriate way to use the stimulants in obesity would be to prescribe for a restricted period only, focusing on the beneficial effects on mood and motivation while establishing the lifestyle changes necessary for weight control, rather than encouraging reliance on the appetite suppression. This would also limit the potential for abuse and adverse long-term cardiovascular effects.

## Psychotropic effects of drugs developed for obesity

Drugs that target hedonic appetite often have actions comparable to the stimulants on mood, motivation, and blood pressure, suggesting a comparable mode of action (Table 1). However, the beneficial psychotropic effects have generally been neglected and instead of dose optimization, studies have tended to randomize participants to fixed doses of active drug or placebo (Munro et al., 1968; Astrup et al., 2008). This methodology means that for some the dose will be too low, while others may have adverse effects on mood due to the dose being excessive. Poor understanding of the need for individual dose titration has resulted in drugs being attributed adverse psychiatric side effects. Given the complexities of the actions of these drugs and their varying affinities for the different neurotransmitter systems, the most practical way to determine the correct dose is by titration to the clinical effects. Titration works well in ADHD where the aim is to improve the functional deficits. Even in obesity there is often scope for improvement in mood and motivation and in our research we have found dose titration possible using adverse effects on mood as an indication for dose reduction (Poulton et al., 2015). Therefore, with correct use the psychotropic effects could have the potential to assist with the lifestyle changes that are vital for weight control. It is important for physicians to understand how best to use these medications (Fujioka, 2015).

**Table 1:** **Drugs that are approved or have been trialed for the treatment of obesity and their psychotropic effects**.

**Mechanism**	**Drug**	**Approved for obesity, either alone, or in combination**	**Favourable psychotropic effects**	**Unfavourable psychotropic effects**	**Euphoria or addiction**
**1. PREVENTING NUTRIENT ABSORPTION**					
Lipase inhibitor	Orlistat	Yes	No	No	
**2. HOMEOSTASIS: ENERGY AND CARBOHYDRATE BALANCE**					
Glucagon-like peptide-1 analog	Liraglutide	Yes	No	No	
**3. CENTRAL EFFECTS**					
Potentiating catecholamines (blocking reuptake, enhancing release)	Stimulants: Phentermine Dexamphetamine	Yes No	Improvements in executive functioning, mood elevation, increased vigor/activity	Anger/hostility, depression, paranoia, hyperlocomotion, psychosis	Yes
	Bupropion	Yes	Improvements in executive functioning	Hyperlocomotion psychosis	Yes
	Tesofensine	No	Mood elevation, increased vigor/activity	Anger/hostility	
Selective serotonin 2C receptor agonist	Lorcaserin	Yes	Reduces impulsive behavior	Fatigue, depression, cognitive impairment	
Cannibinoid type 1 receptor blocker	Rimonabant Taranabant	No No	Increased vigor/activity	Anger/hostility, anxiety, depression, suicide risk	
Mu-opioid receptor antagonist	Naltrexone	Yes	Reduces craving		
Anticonvulsant: mechanism of action in obesity unclear	Topiramate	Yes	Mood improvement	Sedation, cognitive impairment, depression, aggression, psychosis	

*The psychotropic effects may be managed by appropriate dosage adjustments*.

### Phentermine-topiramate

Phentermine has similarities with dexamphetamine in that at low dose a beneficial effect on mood has been observed while higher doses can induce hostility and anger (Brauer et al., 1996). It may be inferred that phentermine, as a stimulant, will lead to dose-related improvements in executive functioning (Gamo et al., 2010) and it has been used effectively for treating ADHD (Rothman, 1996). Although phentermine is considered less addictive than dexamphetamine (Griffiths et al., 1978), it is important to compare its effects at bioequivalent doses. Animal studies have shown that phentermine and dexamphetamine at equipotent doses for appetite suppression are associated with comparable levels of dopamine elevation in the nucleus accumbens (Rowley et al., 2000). Phentermine is generally used in a fixed dose regimen (Munro et al., 1968). It is now available in America in combination with topiramate (United States Food and Drug Administration, 2012; Alfaris et al., 2015) although marketing authority for this combination (Qsymia) was refused by the European Medicines Agency (European Medicines Agency, 2013). This combination uses lower doses of each drug than would be used with either drug given as monotherapy (Alfaris et al., 2015; British National Formulary, 2015). Phentermine is still available in the United Kingdom but is not officially recommended for use in obesity (Kumar and Aronne, 2015).

Topiramate is an anticonvulsant and is associated with sedation and dose related cognitive impairment, causing difficulties with memory and concentration at higher doses (Bray et al., 2003). At low dose it can lead to improvement in mood disorders but at higher doses can be associated with depression, aggression, and psychosis (Besag, 2004). Its mode of action in obesity is unclear, although there may be scope for dose titration to maximize the favorable effect on mood.

### Bupropion-naltrexone

Bupropion is classified as a substituted cathinone, a class of drug that acts as a central nervous system stimulant as it increases the synaptic concentrations of catecholamines by inhibiting reuptake and enhancing their release (Paillet-Loilier et al., 2014). Experimentally in mice bupropion has a similar profile of dopamine related behavioral effects to methamphetamine, being rewarding (reinforcing) at lower doses and causing hyperlocomotion at higher doses (Mori et al., 2013). Interestingly, a study using bupropion for treating obesity documented weight loss with minimal effects on mood or behavior (Anderson et al., 2002), suggesting a low risk of adverse psychotropic effects. Bupropion can improve executive functioning (working memory and sustained attention; Perkins et al., 2013) and has been used for treating ADHD with comparable efficacy to methylphenidate. It has abuse potential, particularly when taken intranasally (Hilliard et al., 2013) and can cause a reversible psychosis (Javelot et al., 2010).

Bupropion is used for obesity in combination with naltrexone (Greenway et al., 2009) a mu opioid receptor antagonist that is used in the treatment of addiction to a range of substances including opiates, nicotine, alcohol and amphetamines as it reduces craving (Woody, 2014). Both bupropion and naltrexone are associated with weight loss and together their effects appear to be synergistic (Greenway et al., 2009). This combination (Contrave) was approved by the FDA for chronic weight management in 2014 (United States Food and Drug Administration, 2014).

### Tesofensine

Tesofensine is a drug that showed efficacy but was abandoned because it caused hypertension (Astrup et al., 2008). Like the stimulants, it blocks the reuptake of dopamine, noradrenaline, and serotonin. Effects on behavior and mood were noted in phase-II studies, with increased activity at all doses and mood changes, particularly at higher doses, including mood elevation and also anger and hostility. That these effects are likely to be dopaminergic is supported by positron emission tomography showing blockade of the dopamine transporter leading to up-regulation of the dopamine pathway (Appel et al., 2014). It can be speculated that as elevated blood pressure was predictable from its mode of action, this might have been managed with lower doses and a more flexible dosing regimen.

### Cannibinoid type 1 receptor blockers rimonabant and taranabant

Rimobanant initially appeared promising as a treatment for obesity as it was associated with improvement in cardiometabolic risk factors that appeared to be in excess of the improvement expected for the degree of weight loss (Nissen et al., 2008). Despite the psychiatric side effects of mood alteration, the drug was approved and marketed. However, once it was being used in greater numbers an association with suicide was observed and all trials were terminated (Topol et al., 2010; McElroy et al., 2015c).

Taranabant is closely related to rimonabant. When rimonabant was withdrawn, all further development of taranabant was terminated (Aronne et al., 2010). In phase-II trials that involved randomization to fixed doses of drug it was noted that psychiatric side effects were the commonest reason for study attrition (Proietto et al., 2010). Anger and hostility were seen at all doses. At the lowest dose there was increased vigor-activity; depression-dejection was seen on the highest dose. These apparently dopaminergic effects may be due to synergy of the dopamine and endocannibinoid pathway (Despres et al., 2005).

## Conclusions

Physicians treating obesity need to understand and appreciate the psychotropic properties of anorexigenic medications, so that treatment can be optimized. The psychiatric side effects that have plagued many efficacy studies might actually be evidence of their mode of action and therefore their efficacy (Astrup et al., 2008; Proietto et al., 2010). This misclassification of the associations of therapeutic efficacy may be sabotaging an important line of drug development. The potential for abuse of drugs with dopaminergic effects is a serious concern but does not preclude their use in ADHD. Indeed there is no clearly apparent logical reason why dexamphetamine is used for treating ADHD while phentermine has been retained for obesity, which raises the question of whether this was simply an accident of history. Ongoing research in our group is examining the effects of dexamphetamine for treating obesity (Poulton et al., 2015). We suggest that the prejudice against the stimulants for treating obesity is perhaps misplaced and favors surgery and new drug development. Meanwhile, in the absence of cheap, effective and accessible treatments the obesity epidemic shows little sign of abating.

## Author contributions

Idea was conceived by AP and RN, who together wrote the first draft. The paper was revised with the input from EH and BC. All authors approved the final version.

## Funding

This paper was unfunded, other than the publication fee, paid by The Nepean Hospital Department of Endocrinology Trust Fund.

### Conflict of interest statement

AP reports personal fees and non-financial support from Shire, outside the submitted work and shares in GSK. BC reports personal fees from Novartis, MSD and Astra Zeneca and non-financial support from Novartis, outside the submitted work. RN reports being director of Vascular Access Technologies Pty Ltd and being listed as one of 6 co-investigators on a provisional patent application lodged through the University of Sydney for a portable automated cannulation device. The other author declares that the research was conducted in the absence of any commercial or financial relationships that could be construed as a potential conflict of interest.
